# First reported survival of anthrax meningoencephalitis in a low-incidence region: successful management with mNGS-guided combination therapy

**DOI:** 10.3389/fcimb.2026.1792720

**Published:** 2026-07-21

**Authors:** Qiushuo Geng, Ye Wang, Yihao Fan, Nan Liu, Xiaoyun Zhao

**Affiliations:** 1 School of Medical Device, Shenyang Pharmaceutical University, Benxi, China; 2 School of Biomedical Engineering, Tsinghua Medicine, Tsinghua University, Beijing, China; 3 Department of Neurology, People’s Hospital of Liaoning Province, Shenyang, China; 4 School of Life Science and Biopharmaceutics, Shenyang Pharmaceutical University, Shenyang, China

**Keywords:** anthrax meningoencephalitis, Bacillus anthracis, metagenomic next-generation sequencing, MNGs, triple antibiotic therapy

## Abstract

We report a rare survival case of anthrax meningoencephalitis in a 56-year-old male from a low-incidence region. The patient presented with nasal discharge, fever, headache, and rapid onset of coma following the slaughter of a diseased cow. Physical examination revealed a characteristic ulcerative eschar on the right index finger, while laboratory investigations showed significant leukocytosis and hemorrhagic cerebrospinal fluid (CSF) characterized by elevated protein and decreased glucose levels. Although initial microscopy misidentified the pathogen as Bacillus cereus, metagenomic next−generation sequencing (mNGS) of the CSF confirmed Bacillus anthracis within 48 hours. This rapid molecular diagnosis enabled a timely switch to a CDC−recommended combination regimen, initially with quadruple therapy (penicillin G, ciprofloxacin, amikacin, and linezolid) followed by optimization to triple therapy (penicillin G, levofloxacin, and linezolid) during the ICU stay, ultimately leading to the patient’s full neurological recovery. This case underscores that the synergistic use of rapid mNGS−based diagnosis and appropriate combination therapy is critical for achieving survival in anthrax meningoencephalitis.

## 1 Introduction

Bacillus anthracis, a Gram-positive spore-forming bacterium, causes anthrax, with anthrax meningoencephalitis representing one of the most lethal complications (Bower et al., 2023). This severe neurological infection occurs in fewer than 5% of all anthrax cases but carries a mortality rate of 80%–100% due to rapid toxin-mediated blood-brain barrier disruption, hemorrhagic inflammation, and subsequent neurological deterioration (Lanska, 2002). The pathogen’s toxins induce endothelial cell apoptosis and inflammatory responses, facilitating bacterial invasion of the central nervous system (Kirby, 2004).

Geographically, anthrax is endemic in livestock-rearing regions, but sporadic cases occur in low-prevalence areas where diagnostic delays are common (Hu et al., 2025). Traditional identification methods face significant challenges: B. anthracis shares morphological and genetic similarities with Bacillus cereus complex species, and culture-based diagnostics require 48–72 hours for results, often leading to misdiagnosis in unfamiliar laboratories (Muigg et al., 2022). Genomic studies have documented that certain B. cereus-group lineages can harbor anthrax toxin genes or anthrax-like virulence determinants, and routine phenotypic or limited molecular assays may misclassify such isolates unless plasmid and virulence gene content are specifically examined (Carroll et al., 2022; Sabin et al., 2024).

In this context, metagenomic next-generation sequencing (mNGS) has emerged as a transformative tool. Systematic reviews show that mNGS applied to cerebrospinal fluid (CSF) increases the breadth of detectable pathogens compared with culture and targeted PCR, with moderate-to-high pooled sensitivity and high specificity; importantly, mNGS often identifies pathogens missed by culture, especially in patients pretreated with antibiotics (Qu et al., 2022; He et al., 2024). While penicillin G remains the first-line treatment for anthrax, combination therapy is recommended for severe cases, and outcomes depend critically on early intervention (Bower et al., 2023). The objective of this report is to demonstrate how rapid mNGS-based diagnosis can guide effective combination therapy for anthrax meningoencephalitis, a condition associated with nearly universal fatality in prior literature. We present a case from a low-incidence region where mNGS enabled timely diagnosis and successful management.

## 2 Methods

### 2.1 Clinical and laboratory evaluation

**Figure 1:**
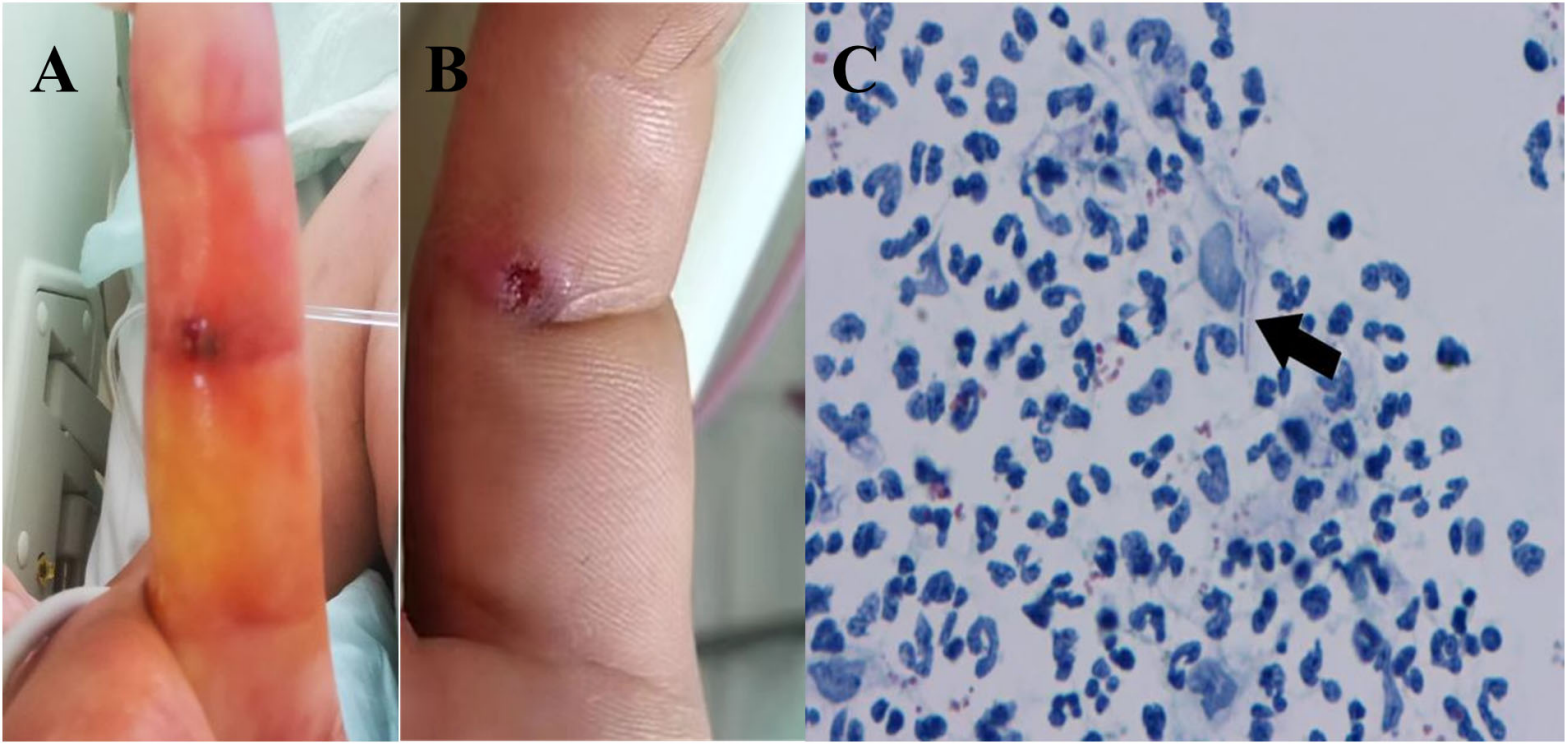
Clinical features of Bacillus anthracis infection. **(A)** Pre-debridement finger wound: A red papule with superficial ulceration and crusting at the volar fissure of the right index finger, without eschar formation. **(B)** Post-debridement finger wound: Clean wound bed with viable granulation tissue and sharp margins, devoid of residual necrosis. **(C)** Cytopathological examination of *B. anthracis* in cerebrospinal fluid (H&E, 10×40): Representative organisms exhibit gram-positive rod morphology in short chains, with bamboo-like segmentation and eosinophilic cytoplasm.

A 56-year-old male patient with suspected severe central nervous system infection was admitted to the People’s Hospital of Liaoning Province. Upon admission, the patient underwent a comprehensive clinical assessment, including vital signs, physical examination with special attention to skin lesions, and neurological examination. Peripheral blood samples were collected for routine hematology, including white blood cell count, neutrophil count, and platelet count; inflammatory markers, including C-reactive protein and procalcitonin; liver and renal function tests; electrolytes; and coagulation parameters, including D-dimer. Lumbar puncture was performed for cerebrospinal fluid (CSF) analysis, including total cell count, white blood cell count, red blood cell count, neutrophil percentage, total protein, chloride, and glucose. Brain magnetic resonance imaging (MRI) was performed to evaluate central nervous system involvement. Direct microscopic examination of CSF was performed for the presence of bacteria. Blood and CSF cultures were conducted according to standard hospital protocols. Cutaneous lesion characteristics were documented photographically (**Figures 1A, B**). Cytopathological analysis of CSF was performed using liquid-based cytology with H&E staining, examined under 400× magnification (**Figure 1C**).

### 2.2 Metagenomic next-generation sequencing

**Figure 2:**
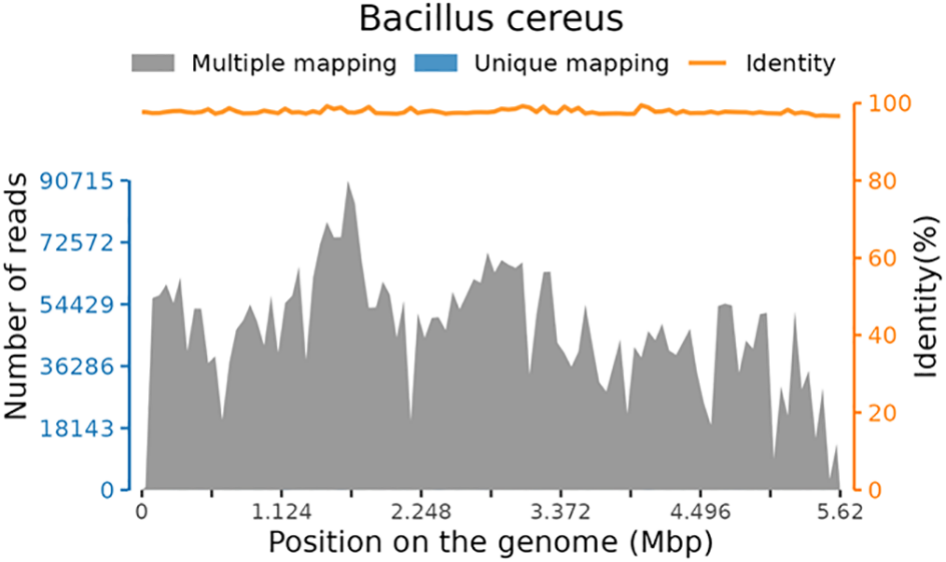
mNGS coverage plot indicating the initial identification of the *Bacillus cereus* group. The x-axis represents the physical position on the reference genome (Mbp), and the y-axis indicates the number of sequencing reads (left) and sequence identity percentage (right, orange line). The overwhelming dominance of multiple mapping reads (grey area) reflects the extremely high genomic homology within the *B. cereus* sensu lato complex, resulting in a group-level taxonomic assignment by the automated pipeline.

**Figure 3:**
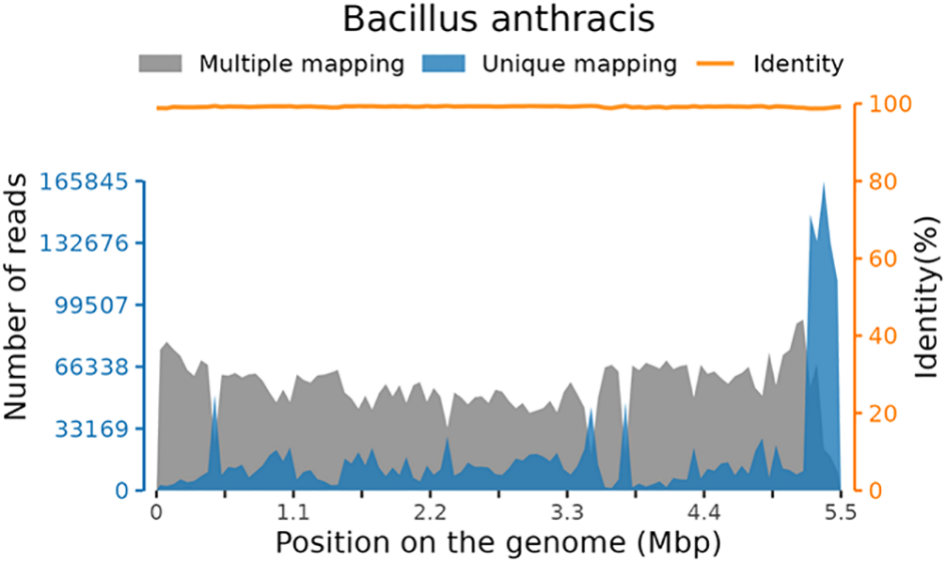
Targeted mNGS coverage plot confirming *Bacillus anthracis* via specific virulence loci. Axes definitions follow **Figure 2**. Following targeted bioinformatic re-analysis, a distinct accumulation of unique mapping reads (blue area, 4.8–5.5 Mbp) is evident. These uniquely mapped sequences correspond to anthrax-specific virulence plasmids (pXO1/pXO2), resolving the prior taxonomic ambiguity and establishing a definitive species-level diagnosis.

On hospital day 2, a CSF sample was collected and sent to KingMed Diagnostics (Shenyang, China) for mNGS testing. Sequencing was performed on the MGISEQ-200 platform. The sequencing generated a total of 14,597,775 reads, with a host read proportion of 1.44%. The remaining non-host reads were taxonomically classified against microbial reference databases. Initial taxonomic classification assigned 7,431,844 non-host reads to the *Bacillus cereus* group (**Figure 2**). Because the *B. cereus* group includes multiple species with high genomic homology, a targeted bioinformatic re-analysis was subsequently performed. This re-analysis specifically queried the presence of the *Bacillus anthracis*-specific virulence plasmids pXO1 and pXO2, confirming their presence (**Figure 3**).

### 2.3 Antibiotic susceptibility testing

Antibiotic susceptibility testing was performed on cultured isolates according to standard methods. Antibiotic susceptibility testing indicated that the isolate was sensitive to multiple agents but resistant to trimethoprim-sulfamethoxazole.

### 2.4 Treatment protocol and adjustments

The patient’s clinical course and therapeutic interventions are summarized chronologically in **Table 1**. Upon admission, empirical intravenous meropenem (2.0 g every 8 hours) was initiated along with dexamethasone (10 mg daily). On hospital day 2, following clinical deterioration and detection of Gram-positive bacilli on CSF microscopy, vancomycin (0.5 g every 6 hours) and ciprofloxacin (0.4 g every 12 hours) were added to meropenem, establishing a broad-spectrum triple-agent regimen.

**Table 1:** Chronological clinical course and therapeutic interventions.

Time	Patient symptoms and clinical findings	Therapeutic schedule
2 days before admission	Assisted in slaughtering a diseased cow; a pre-existing small wound was present on the inner side of the right index finger. Developed nasal discharge.	Intravenous ribavirin, clindamycin, ambroxol, and levofloxacin were administered at a local hospital.
Day 1 (Admission)	Fever (39.6 °C), headache, altered consciousness. Physical examination revealed a 0.5 × 0.5 cm erythematous papule with ulceration and scab formation on the right index finger. Peripheral blood tests showed elevated WBC (19.7 × 10^9^/L) and CRP (71.69 mg/L). CSF analysis revealed markedly elevated total cell count and WBC, increased RBC count, elevated protein (4.21 g/L), and reduced glucose (2.94 mmol/L), suggesting hemorrhagic bacterial meningitis.	Empirical intravenous meropenem (2.0 g q8h) initiated; dexamethasone (10 mg/day) administered.
Hospital day 2	Neurological deterioration to moderate-to-severe coma, with respiratory distress and limb convulsions. Peripheral WBC increased to 20.96 × 10^9^/L. Repeat CSF analysis showed further elevation of WBC and RBC counts, increased protein (5.78 g/L), and glucose 3.42 mmol/L. Gram-positive bacilli observed on direct CSF microscopy.	Vancomycin (0.5 g q6h) and ciprofloxacin (0.4 g q12h) added to meropenem. A CSF sample was collected and sent for mNGS testing.
Hospital day 3	Persistent coma and fever despite partial decline in peripheral WBC (15.08 × 10^9^/L). Procalcitonin was markedly elevated (18.1 ng/mL). Laboratory evidence of hepatic dysfunction, including elevated ALT (112.7 U/L). mNGS of CSF identified *Bacillus cereus* group.	Current antimicrobial regimen maintained while awaiting confirmatory results.
Hospital day 4	Genetic sequencing and confirmatory testing of blood, CSF, and skin samples identified *Bacillus anthracis*, establishing the diagnosis of cutaneous anthrax with anthrax meningoencephalitis. Respiratory failure progressed.	Antimicrobial regimen adjusted according to CDC guidelines: high-dose penicillin G (8 million U q6h), ciprofloxacin (0.4 g q12h), amikacin (0.5 g q12h), and linezolid (600 mg q12h). Endotracheal intubation and mechanical ventilation were initiated. The patient was transferred to an infectious disease hospital ICU.
ICU day 1-4	Patient remained comatose with a persistent high fever.	Regimen was adjusted by the receiving ICU team to dual therapy: penicillin G (8 million U q6h) and levofloxacin (0.5 g q12h).
ICU day 5 to Ward discharge	Consciousness gradually improved, and the fever resolved.	Linezolid (600 mg q12h) was re-added per CDC guidelines to suppress toxin production. Maintained on this triple therapy for 17 days.
1.5-year follow-up	Patient is fully conscious with normal neurological function. Follow-up lumbar puncture showed clear CSF with normalized pressure.	No further antimicrobial treatment required.

WBC, white blood cell count; CRP, C-reactive protein; CSF, cerebrospinal fluid; RBC, red blood cell; ALT, alanine aminotransferase; mNGS, metagenomic next-generation sequencing; ICU, intensive care unit; CDC, Centers for Disease Control and Prevention.

On hospital day 4, mNGS and confirmatory testing by the local CDC established the diagnosis of anthrax meningoencephalitis. The patient had cutaneous anthrax with central nervous system involvement. Based on the 2005 China CDC and 2014 US CDC guidelines, the antibiotic regimen was adjusted accordingly (Ministry of Health of the People's Republic of China, 2005; Hendricks et al., 2014). The regimen comprised high-dose penicillin G (8 million U every 6 hours), ciprofloxacin (0.4 g every 12 hours), amikacin (0.5 g every 12 hours), and linezolid (600 mg every 12 hours). That evening, due to worsening respiratory failure, the patient was intubated and placed on mechanical ventilation, then transferred to the intensive care unit (ICU) of a specialized infectious disease hospital on day 6.

Upon admission to the receiving ICU, the antimicrobial regimen was initially adjusted to dual therapy (penicillin G 8 million U q6h and levofloxacin 0.5 g q12h) for four days. However, because the patient remained comatose with persistent high fever, linezolid (600 mg q12h) was reintroduced in accordance with the 2014 US CDC guidelines recommending a protein synthesis inhibitor to suppress toxin production. The patient received this optimized triple regimen (penicillin G, levofloxacin, and linezolid) for 17 days. After discharge, the patient completed an oral quadruple antimicrobial regimen for four months. Antimicrobial therapy was discontinued only after CSF white blood cell count normalized and two consecutive follow-up mNGS tests confirmed clearance of *B. anthracis*. The patient also received oral prednisone for one month to manage residual neuroinflammation and persistent headaches.

## 3 Results

### 3.1 Patient baseline and exposure history

The patient was a 56-year-old male. Two days before symptom onset, he had assisted in slaughtering a diseased cow. The animal owner subsequently died from a fatal infection, and two other contacts developed skin lesions. Prior to the exposure, the patient had a small wound on the inner side of his right index finger (**Figure 1A**). Two days before admission, he received intravenous ribavirin, clindamycin, ambroxol, and levofloxacin at a local hospital. On the day of admission, head and chest computed tomography (CT) at the local hospital showed no abnormalities.

### 3.2 Admission clinical findings

Upon admission, the patient was confused, restless, and able to open and close his eyes spontaneously but did not respond to verbal stimuli. Physical examination revealed a body temperature of 39.6 °C, pulse rate of 114 beats per minute, respiratory rate of 34 breaths per minute, and blood pressure of 147/91 mmHg. A 0.5 cm × 0.5 cm erythematous papule with ulceration and scab formation was noted on the inner side of his right index finger (**Figure 1B**). No blistering or superficial lymphadenopathy was observed. Cardiopulmonary and abdominal examinations were unremarkable. Neurological examination showed an altered mental status with absent verbal responses. Pupils were equal, round, and reactive to light (diameter ~3 mm). Facial symmetry was preserved. Limb movements were preserved, and deep and superficial reflexes were symmetrically present. Meningeal irritation signs were positive, and bilateral pathological reflexes were positive. Coordination and sensory examination were inconclusive due to poor patient cooperation.

### 3.3 Initial laboratory and imaging results

Peripheral blood tests showed elevated white blood cell count (WBC 19.7×10^9^/L) and neutrophil count (18.14×10^9^/L). C-reactive protein (CRP) was 71.69 mg/L, and procalcitonin was 0.172 ng/mL. D-dimer was 6.17 mg/L. Liver function and electrolytes were approximately normal. Blood glucose was 7.84 mmol/L. CSF analysis revealed a total cell count of 3813×10^6^/L, WBC count of 1813×10^6^/L, red blood cell (RBC) count of 2000×10^6^/L, neutrophil percentage of 81.9%, total protein of 4.21 g/L, chloride of 116 mmol/L, and glucose of 2.94 mmol/L. Brain MRI showed linear hyperintensities along the cerebral sulci on T2 FLAIR imaging. Based on the history, presentation, and laboratory findings, bacterial hemorrhagic meningitis was suspected.

### 3.4 Clinical deterioration on hospital day 2

On hospital day 2, the patient’s condition worsened to moderate-to-severe coma, with respiratory distress and limb convulsions. Repeat blood tests showed WBC 20.96×10^9^/L and neutrophils 19.22×10^9^/L. Repeat CSF analysis showed total cell count 7361×10^6^/L, WBC 4361×10^6^/L, RBC 3000×10^6^/L, neutrophil percentage 96.5%, total protein 5.78 g/L, chloride 115 mmol/L, and glucose 3.42 mmol/L. Direct microscopic examination of CSF revealed large Gram-positive bacilli.

### 3.5 mNGS and diagnostic confirmation (Days 3–4)

On hospital day 3, mNGS of the CSF identified the Bacillus cereus group (**Figure 2**). Concurrently, blood and CSF cultures yielded Gram-positive bacilli. Liquid-based cytopathological analysis of CSF showed macrophages containing intracellular bacilli (**Figure 1C**). Antibiotic susceptibility testing indicated sensitivity to multiple agents except trimethoprim-sulfamethoxazole (resistant). Despite a decline in WBC (15.08×10^9^/L) and neutrophils (12.83×10^9^/L), procalcitonin rose to 18.1 ng/mL, and the patient remained in moderate-to-severe coma with persistent fever, type I respiratory failure, hepatic dysfunction (ALT 112.7 U/L, GGT 863 U/L), hypoalbuminemia (32.6 mg/dL), and electrolyte imbalances.

On hospital day 4, targeted bioinformatic re-analysis confirmed the presence of pXO1 and pXO2 virulence plasmids, establishing the diagnosis of B. anthracis (**Figure 3**). Subsequent testing of blood, CSF, and skin samples by the CDC confirmed anthrax. The patient was diagnosed with cutaneous anthrax and anthrax meningoencephalitis.

### 3.6 Treatment response and ICU course

Following diagnosis, the antibiotic regimen was adjusted to CDC-recommended quadruple therapy (penicillin G, ciprofloxacin, amikacin, and linezolid). That evening, due to worsening respiratory failure, the patient was intubated and placed on mechanical ventilation, then transferred to an infectious disease hospital ICU on day 6. In the receiving ICU, the regimen was initially changed to dual therapy (penicillin G and levofloxacin) for four days, but the patient remained comatose with persistent high fever. Linezolid was reintroduced, forming a triple regimen (penicillin G, levofloxacin, and linezolid). The patient showed progressive neurological improvement on this regimen.

### 3.7 Outcome and follow-up

After successful weaning from mechanical ventilation, the patient completed a 17-day course of triple therapy, followed by four months of oral quadruple antimicrobial therapy. Antimicrobial therapy was discontinued only after CSF WBC normalized and two consecutive follow-up mNGS tests confirmed clearance of B. anthracis. He also received oral prednisone for one month for residual neuroinflammation and headaches. At present, the patient is conscious and responsive, afebrile, seizure-free, with normal limb mobility, though occasional headaches and head pressure persist. A follow-up lumbar puncture 1.5 years after disease onset showed clear CSF with an opening pressure of 200 mmH_2_O. The patient remains asymptomatic with no evidence of recurrence. The detailed chronological clinical course and therapeutic interventions are summarized in **Table 1**.

## 4 Discussion

We report the first survival of anthrax meningoencephalitis diagnosed by metagenomic next-generation sequencing (mNGS) in a low-incidence region. The patient’s complete neurological recovery contrasts with the historically near-universal fatality of this condition (Lanska, 2002; Bower et al., 2023).

### 4.1 Diagnostic value of mNGS.

Conventional microscopy and culture initially misidentified the pathogen as *Bacillus cereus*, a common diagnostic pitfall due to high genomic homology within the *B. cereus* group. mNGS correctly assigned the reads to the *B. cereus* group, but, crucially, targeted re-analysis for virulence plasmids pXO1 and pXO2 enabled definitive species-level identification of *B. anthracis* within 48 hours. This capability is particularly valuable in low-incidence regions where clinical suspicion is low and conventional diagnostics may be delayed (Muigg et al., 2022). Previous mNGS-diagnosed cases of anthrax meningoencephalitis were fatal (Hu et al., 2025); our case demonstrates that rapid molecular diagnosis can change the outcome when combined with timely therapy.

### 4.2 Validation of CDC-recommended combination therapy.

Current CDC guidelines recommend that suspected anthrax meningitis be treated with at least three antimicrobials, including a protein synthesis inhibitor (PSI) such as linezolid or clindamycin (Bower et al., 2023). This recommendation is based largely on animal studies and theoretical reasoning because human survivorship data are scarce (Holty et al., 2006). Our case provides rare clinical evidence supporting this approach. After initial improvement on quadruple therapy (penicillin G, ciprofloxacin, amikacin, linezolid), the patient was switched to dual bactericidal therapy (penicillin G + levofloxacin). He remained comatose with persistent fever until linezolid was reintroduced, after which progressive neurological recovery occurred. This temporal association supports the PSI’s role in toxin suppression, consistent with *in vitro* data showing that linezolid maintains *B. anthracis* in the vegetative stage, in which toxins are produced, but inhibits toxin synthesis, whereas bactericidal agents alone may leave the host vulnerable to ongoing toxin−mediated damage (Louie et al., 2012). Recent pharmacokinetic modeling further explains why amikacin, with its low CSF penetration, may be dispensable, while linezolid, with its modest CSF penetration, remains critical for its virulence−modulating effect (Bradley et al., 2024).

### 4.3 Clinical implications.

For clinicians in non-endemic areas, a history of livestock exposure, a cutaneous eschar, and rapid neurological deterioration should prompt immediate consideration of anthrax meningoencephalitis. Early CSF mNGS with targeted plasmid analysis can resolve taxonomic ambiguity and guide life-saving combination therapy.

## Data Availability

The host-depleted metagenomic sequencing data have been deposited in the Genome Sequence Archive (GSA) at the China National Center for Bioinformation (CNCB) under accession number CRR2900451 (Project PRJCA061663, Sample SAMC7447983), that are publicly accessible at https://ngdc.cncb.ac.cn/gsa/browse/CRA041079.
